# Genomic Sequencing and Analysis of Eight Camel-Derived Middle East Respiratory Syndrome Coronavirus (MERS-CoV) Isolates in Saudi Arabia

**DOI:** 10.3390/v12060611

**Published:** 2020-06-03

**Authors:** Badr M. Al-Shomrani, Manee M. Manee, Sultan N. Alharbi, Mussad A. Altammami, Manal A. Alshehri, Majed S. Nassar, Muhammed A. Bakhrebah, Mohamed B. Al-Fageeh

**Affiliations:** 1 National Centre for Biotechnology, King Abdulaziz City for Science and Technology, Riyadh 11442, Saudi Arabia; snharbi@kacst.edu.sa (S.N.A.); mtammami@kacst.edu.sa (M.A.A.); Manalalshehri@kacst.edu.sa (M.A.A.); mnassar@kacst.edu.sa (M.S.N.); mbakhrbh@kacst.edu.sa (M.A.B.); mfageeh@kacst.edu.sa (M.B.A.-F.); 2 Institute of Bioinformatics, University of Georgia, Athens, GA 30602, USA

**Keywords:** dromedary camel, MERS-CoV, 2019-nCov, phylogenetic analysis, vaccine design

## Abstract

Middle East respiratory syndrome coronavirus (MERS-CoV) causes severe respiratory illness in humans; the second-largest and most deadly outbreak to date occurred in Saudi Arabia. The dromedary camel is considered a possible host of the virus and also to act as a reservoir, transmitting the virus to humans. Here, we studied evolutionary relationships for 31 complete genomes of betacoronaviruses, including eight newly sequenced MERS-CoV genomes isolated from dromedary camels in Saudi Arabia. Through bioinformatics tools, we also used available sequences and 3D structure of MERS-CoV spike glycoprotein to predict MERS-CoV epitopes and assess antibody binding affinity. Phylogenetic analysis showed the eight new sequences have close relationships with existing strains detected in camels and humans in Arabian Gulf countries. The 2019-nCov strain appears to have higher homology to both bat coronavirus and SARS-CoV than to MERS-CoV strains. The spike protein tree exhibited clustering of MERS-CoV sequences similar to the complete genome tree, except for one sequence from Qatar (KF961222). B cell epitope analysis determined that the MERS-CoV spike protein has 24 total discontinuous regions from which just six epitopes were selected with score values of >80%. Our results suggest that the virus circulates by way of camels crossing the borders of Arabian Gulf countries. This study contributes to finding more effective vaccines in order to provide long-term protection against MERS-CoV and identifying neutralizing antibodies.

## 1. Introduction

Coronaviruses are a family of single-stranded, positive-sense RNA genomes that range from 26,000 to 32,000 basepairs in length. This family can infect both humans and animals, and has been found in many mammal hosts, including bats, camels, and pigs [1]. Coronaviruses are also known to have high genetic diversity influenced by mutation and recombination, which can lead to the development of new viruses. Before 2003, human coronaviruses were primarily known for causing common colds. In 2003, severe acute respiratory syndrome coronavirus (SARS-CoV) was first diagnosed in southern China as an epidemic, and went on to infect almost 8000 people in 29 countries, with a fatality rate of approximately 10% [2,3]. Almost ten years later, in 2012, another novel and deadly human coronavirus emerged, named the Middle East respiratory syndrome coronavirus (MERS-CoV); it infected more than 1700 people and exhibited a high mortality rate of ∼36% [4,5]. On 30 December 2019, a third coronavirus outbreak emerged in Wuhan, China, designated as the 2019 novel coronavirus (2019-nCoV), which is still ongoing and has now spread over 213 countries, becoming a pandemic. According to the daily report of the World Health Organization (WHO) on 17 April 2020, 1,995,983 confirmed cases and 131,037 deaths worldwide are attributed to the pandemic so far. These novel betacoronaviruses are global threats with the potential for further outbreaks that cause substantial health and economic consequences. Better understanding of their evolutionary history is necessary in order to determine their origin and spread and to effectively design interventions and neutralizing antibodies.

Of the betacoronaviruses that have caused major outbreaks to date, MERS-CoV is notable for having the highest mortality rate and for being centered in the Middle East. It was first detected in Saudi Arabia in 2012, in a patient with a fatal respiratory infection [6]. The viral respiratory illness caused by MERS-CoV is known as Middle East respiratory syndrome (MERS), and its symptoms range from fever, cough, and shortness of breath to severe illness and respiratory failure. The symptoms typically develop within 2–14 days, but some patients do not demonstrate any clinical symptoms despite testing positive for MERS-CoV in a laboratory assay. Severe symptoms are more likely to appear in older people, those with weakened immune systems, and those with chronic diseases such as cancer or diabetes. Since the initial identification of MERS, Saudi Arabia has continued to report the largest number of cases. As of February 2019, 1983 cases and 783 associated deaths have been detected in Saudi Arabia, producing a case fatality rate of 35.7% [7].

The exact origin of MERS-CoV is still uncertain, and further studies remain necessary to confirm the source of its transmission to humans [8]. Notably, Arabian camels (*Camelus dromedarius*) from Saudi Arabia and other Gulf countries are found to be seropositive for MERS-CoV at high rates [9,10]. Furthermore, a retrospective study carried out on archived sera of dromedary camels from 1983 to 1997 revealed neutralizing antibodies whose presence implies a long-term circulation of MERS-CoV among Arabian camels [11]. These camels are thought to be an intermediate host for the virus, with its ancestral origin being associated with African bats [12,13]. Furthermore, data from many investigations about the MERS-CoV outbreak suggests that camels could be a source of human infections [14,15,16]. *C. dromedarius* is a member of the camelid family, plays a significant role in transportation, is a source of essential products such as meat and milk [17], and is overall a central part of the heritage and way of life of most residents of Arabian Peninsula countries [18]. Although many MERS-CoV patients had no direct contact with camels [19,20], an indirect path of infection has been proposed through camel workers, who have direct and prolonged contact. Additionally, travelers who visit the Middle East are vulnerable to infection and may seed the virus in other regions, including Europe, North America, and Southeast Asia [21]; for example, phylogenetic analysis of a South Korean outbreak in 2015 traced its evolutionary history to Saudi Arabian strains [22]. Accordingly, in-depth genetic analysis of strains within Saudi Arabia would provide an essential reference and resource in the face of further outbreaks. Of particular research interest is the MERS-CoV spike protein, which enables virus entry into host cells via binding to cellular receptor dipeptidyl peptidase-4 (DPP4), and therefore is considered a key target for vaccine design [23].

This study aimed to analyze the evolutionary history of eight newly sequenced MERS-CoV isolated from dromedary camels in Saudi Arabia, along with representative members of the genus Betacoronavirus, based on both complete genomes and spike protein sequences. We applied the time-framed Bayesian evolution analysis approach implemented in BEAST to determine the evolutionary relationships between these sequences. We also utilized the Immune Epitope Database and Analysis Resource (IEDB) to collect known epitope sites from other betacoronaviruses, map equivalent protein sites in MERS-CoV, and predict likely epitopes. This study provides insights into the origin of MERS-CoV and likely means of transmission through Gulf countries.

## 2. Results

### 2.1. Genome Organization

The consensus MERS-CoV genome obtained through analyzing isolates from eight infected camels from different locations across Saudi Arabia was 30,118 nt in length with a GC content of 41%. The eight constituent genomes shared >99% identity. The camel MERS-CoV genome is similar to that of human MERS-CoV, containing ten ORFs (ORF1ab, ORF3, spike [S], ORF5, ORF4a, ORF4b, envelope [E], membrane [M], nucleocapsid [N], and ORF8b). BLAST comparison to other camel and human MERS-CoV strains available from NCBI revealed that all share ∼99% identity with one another. The comparative analysis of homologous sequences of spike proteins and genes has been performed for the 31 Betacoronavirus strains (Table 1). The MERS-CoV strains were nearly identical across their spike proteins and genes (>99.50% and >81%, respectively). Indicative of a very recent emergence into the human population.

### 2.2. Phylogenetic Analysis

**Figure 1:**
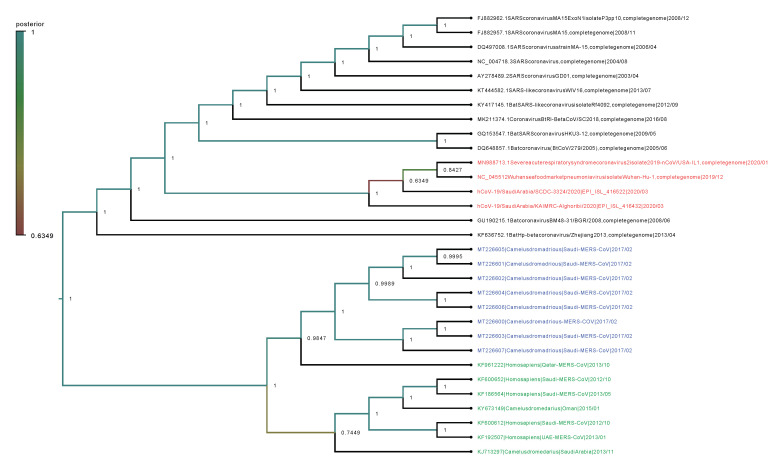
Phylogenetic analysis of the complete genomes of our eight MERS-CoV isolates and representative viruses of the genus Betacoronavirus. The new eight isolates are in blue, other MERS-CoV strains in green, and 2019-nCoV in red. The numbers at nodes represent the posterior probabilities of their clustering.

**Figure 2:**
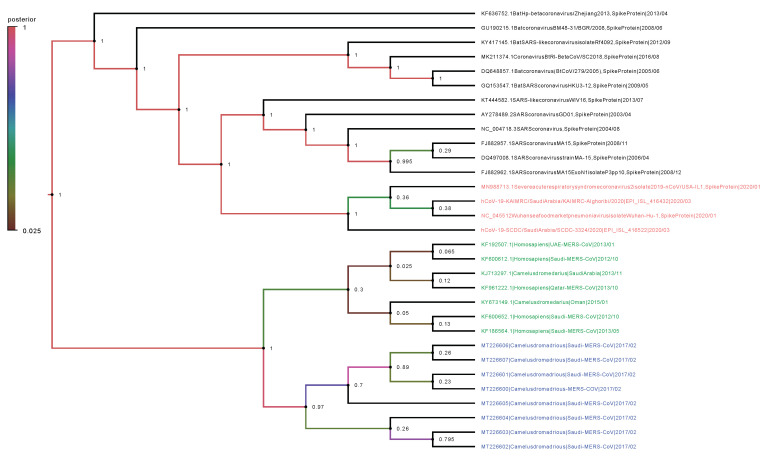
Phylogenetic analysis of spike proteins of our eight MERS-CoV isolates and representative viruses of the genus Betacoronavirus. The new eight isolates are in blue, other MERS-CoV strains in green, and 2019-nCoV in red. The numbers at nodes represent the posterior probabilities of their clustering.

Phylogenetic analysis was performed on the complete genomes and spike proteins of the eight new MERS-CoV sequences and 23 representative viruses from the genus Betacoronavirus. The eight camel-derived MERS-CoV sequences clustered in a distinct clade and were all relatively close to other MERS-CoV sequences from human and camel hosts (Figure 1). The 2019-nCoV falls in a distinct clade and shows a closer relationship to Bat-SARS-Like (GU190215.1) than the MERS-CoV strains. In agreement with the complete genome results, analysis based on the spike proteins showed that 2019-nCoV has a closer relationship to Bat-SARS-Like and SARS-CoV strains than MERS-CoV strains (Figure 2).

We observed very high conservation between the new eight sequenced MERS-CoV genomes, with sequence identity >99%, despite the different geographical locations of sampling. In addition, a specific sample collected in 2015 from a human in Qatar was revealed to be more closely related to the eight new MERS-CoV isolates than to other UAE and Oman MERS-CoV strains. Genetically, the MERS-CoV genome can overall be considered more diverse than those of other betacoronavirus family members. Phylogenetic analysis of the MERS-CoV spike protein and its counterparts showed that the four clades formed four well-supported branches (Figure 2).

### 2.3. MERS-CoV B Cell Epitope Analysis

**Figure 3:**
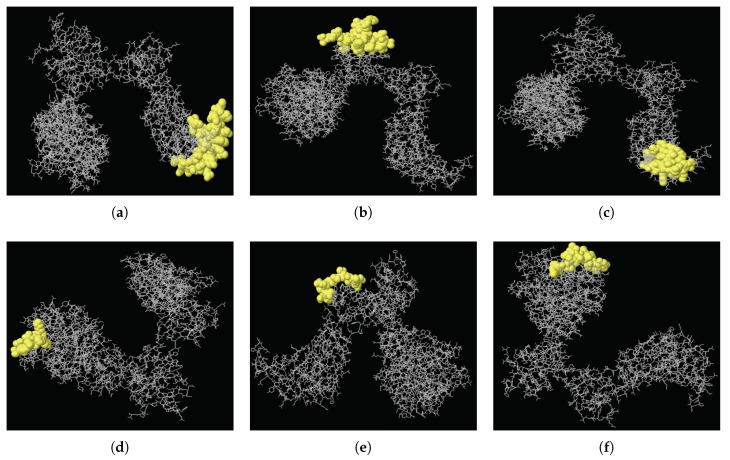
Three-dimensional locations of discontinuous epitopes (**a**–**f**) predicted in the MERS-CoV spike protein. The epitopes are shown in yellow, with the bulk of the MERS-CoV spike protein as grey sticks.

B cell epitopes are significant in the defense of the immune system against viral diseases, allowing B cells to identify different viral infections and activate responses against them. There has been many studies aiming to design MERS-Cov vaccine [24,25,26]. In this study, epitope analysis of MERS-CoV showed that the MERS-CoV spike protein has 24 discontinuous regions in total, from which just six epitopes with score values of >0.8 could be selected. The higher the score, the greater the potential of a true discontinuous epitope; the highest probability obtained was 0.898. Table 2 details the peptides of these high-scoring discontinuous epitopes and their sequence locations, epitope lengths, and associated scores. Their positions on the 3D structure of the MERS-CoV spike protein (PDB ID: 5X5F) are illustrated in Figure 3.

## 3. Discussion

MERS-CoV is a zoonotic virus for which ongoing studies indicate major roles in transmission for direct contact with live camels and with humans having symptomatic MERS [27,28,29], with dromedary camels being considered intermediate hosts that are the main source of human infections. The second largest outbreak to date occurred in Saudi Arabia, and MERS-CoV is widespread among humans and camels in the Gulf countries [30], where it remains a potential hazard to regional and global health and welfare. The present study seeks to determine the evolutionary relationships between eight new MERS-CoV isolates obtained from Arabian camels in different regions of Saudi Arabia and 23 other previously sequenced betacoronavirus strains, based on phylogenetic trees constructed with BEAST for the complete viral genome and the spike protein.

In the phylogenetic analysis, the eight new strains clustered in a separate clade, exhibiting close relationships with strains obtained from MERS-CoV-infected camels and humans in Saudi Arabia, the United Arab Emirates (UAE), Qatar, and Oman. This finding supports the expectation that the virus circulates via camels crossing borders with Oman and other Gulf countries, and is also consistent with the report of another study that movement of camels between Saudi Arabia, the UAE, and Oman contributes to the spread of the virus throughout the Arabian Peninsula [31]. Based on these results, it is recommended that restrictions be implemented to prevent these animals from migrating between Gulf countries.

Within the Betacoronavirus genus, SARS-CoV and MERS-CoV are distinct from 2019-nCoV [32]; the 2019-nCoV genome and spike glycoprotein, respectively, showed 48.83% and 41.95% sequence identity with human-derived MERS-CoV [33]. The 2019-nCoV virus was found to be most closely related to Bat-SARS-Like [1]. This confirms the identification of bats as reservoirs for coronaviruses, including MERS-CoV and 2019-nCoV.

In conclusion, this study determined the evolutionary relationships of newly sequenced MERS-CoV, along with 23 previously sequenced isolates of the genus Betacoronavirus. We also performed genomic characterization of MERS-CoV, highlighting its origin and likely means of transmission through Gulf countries. In addition, we analyzed the MERS-CoV spike protein for suitable epitopic candidates and identified six viral peptides with good antigen presentation scores (>80%).

## 4. Materials and Methods

### 4.1. Sample Collection

The samples were collected from 30 dromedary camels at different locations in Saudi Arabia, following the guidelines of the Food and Agriculture Organization of the United Nations. The camel was appropriately restrained, and the camel nasal wing was then held and raised by the thumb and index finger, followed by gentle insertion of the nasal swab by the upper half of the nasal vestibule as deep as possible until the swab is handled. The swab was then laterally rotated, transferred into a tube containing virus transport media, and kept at 4 °C in preparation for PCR screening.

### 4.2. RNA Extraction

Total RNA was successfully extracted from eight samples of camel nasal fluids using a MagMax-96 viral RNA extraction kit (Thermo Fisher Scientific, Waltham, MA, USA) as per the manufacturer’s protocol. The final DNAase-treated RNA product was stored at −80 °C.

### 4.3. Sequence Preparation

**Figure 4:**
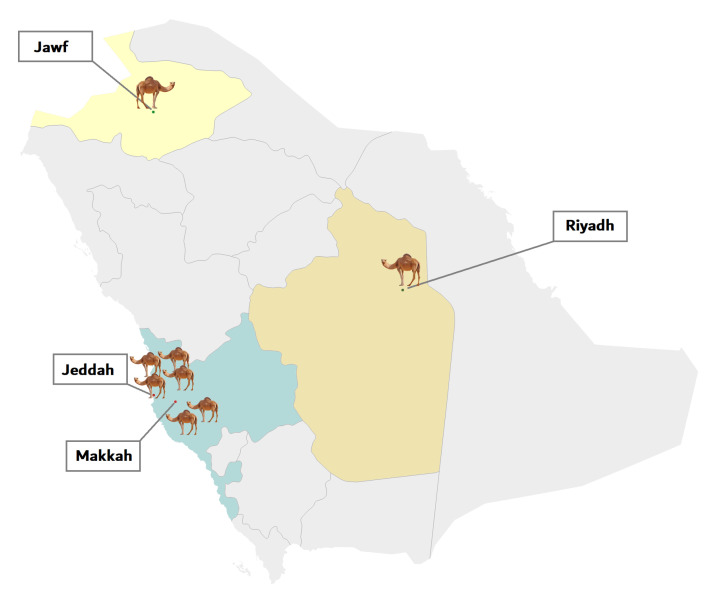
Geographical distribution of dromedaries in Saudi Arabia infected with MERS-CoV viruses that were isolated in the present study. The number of dromedaries corresponds to the number of isolates from the sampling location.

Eight complete genomes of MERS-CoV from *C. dromedarius* have been sequenced in our lab. The samples came from multiple geographical locations in Saudi Arabia: four from Jeddah city, one from Northern Saudi Arabia, two from Mecca city, and one from Riyadh city (Figure 4). For the sake of comparison, we also retrieved 21 complete genomes of betacoronavirus strains from the National Center for Biotechnology Information (NCBI) database (http://www.ncbi.nlm.nih.gov/genomes/VirusVariation/Database/nph-select2.cgi), along with those of two 2019-nCoV strains isolated in Saudi Arabia (https://www.gisaid.org). In total, 31 betacoronavirus sequences were analyzed (Table 3).

### 4.4. RNA Quantification and cDNA Conversion

The concentration of each RNA sample was measured on a Qubit 3.0 using the Qubit RNA BR assay kit (Thermo Fisher Scientific, Waltham, MA, USA) and reverse transcribed with a SuperScript II cDNA kit (Thermo Fisher Scientific, Waltham, MA, USA). A minimum RNA concentration of 100 ng/uL was used for cDNA conversion.

### 4.5. Library Preparation and Sequencing

High-quality cDNA was subjected to Ampliseq library preparation as per the manufacturer’s protocol using MERS Ampliseq Panels (Thermo Fisher Scientific, Waltham, MA, USA) with a mean insert size of 200 bp. Each library was assigned to a distinct barcode using the Ion Xpress Barcode Adapters 1–16 kit and purified using Agencourt AMpure XP beads (Beckman Coulter, Brea, CA, USA). Purified libraries were efficiently quantified on a StepOnePlus Real-Time PCR system using an Ion Library TaqMan quantification kit (Thermo Fisher Scientific, Waltham, MA, USA) as per the manufacturer’s protocol. Libraries were normalized to 25 pM, and template-positive Ion Sphere particles (ISPs) were obtained by pooling all ten normalized libraries and performing clonal amplification on a OneTouch 2 system using the Ion PGM HiQ OT2 200 kit (Thermo Fisher Scientific, Waltham, MA, USA). Template-positive ISPs were then loaded on Ion 318 chips and sequenced on an Ion PGM instrument using the Ion PGM HiQ Sequencing kit (Thermo Fisher Scientific, Waltham, MA, USA). The resulting data were preprocessed (base calling, base quality recalibration, alignment and consensus sequence assembly) using Torrent Suite Server v 5.6. Ninety percent of the consensus sequences were obtained from this data; the remaining gaps were filled by Sanger sequencing on a 3730xl Genetic Analyzer using custom-designed primers (Macrogen LLC, Seoul, Korea) and BigDye Terminator v 3.1 Cycle Sequencing chemistry (Thermo Fisher Scientific, Waltham, MA, USA).

### 4.6. Gap Filling and Sanger Sequencing

To fill the gaps that remained after mapping Ion reads, we designed 11 primer sets (Table 4) against the NCBI MERS-CoV reference genome assembly (NC_019843.3). Primers were designed and checked using Primer Express software (v3.0) (Applied Biosystems, Foster City, CA, USA) and NetPrimer (Premier Biosoft, Palo Alto, CA, USA), with gap flanking regions incorporated into the primers. Sequences for all the major gaps were amplified by PCR using Platinum^TM^ Hot Start PCR Master Mix 2X (Thermo Fisher Scientific, Waltham, MA, USA). PCR products were sequenced using the BigDye^®^ Terminator Sequencing Kit (Applied Biosystems, Foster City, CA, USA) on an Applied Biosystems 3730xl DNA Analyzer. Most minor gaps were sample-specific; to ensure complete genome coverage for phylogenetic analysis, these gaps were closed manually after performing multiple sequence alignment (MSA) on the fully-sequenced genomes. MSA was carried out using Geneious software [34].

### 4.7. Phylogenetic Trees and Evolutionary Dynamics

We first performed multiple sequence alignment of the 31 strains using MAFFT (v7) [35]. Next, we estimated the phylogenetic relationships using a time-framed Bayesian evolution analysis approach via a Markov Chain Monte Carlo (MCMC) inference method; this approach was implemented in the BEAST package (v1.8.2) [36]. For analysis of whole genomes, we used the GTR+I substitution model, a strict clock model, and a constant-size tree prior. For the spike protein dataset, we used the Blosum62+Gamma substitution model, a strict clock model, and a constant-size tree prior. MCMC analyses were run for 20 million iterations, sampling every 100 thousand iterations after a 10% burn-in. We assessed each run’s convergence using Tracer (v1.6) [37]. The BEAST package TreeAnnotator was utilized to summarize a maximum clade credibility (MCC) tree for every dataset using MCMC tree samples. Phylogenetic clustering patterns were visualized and analyzed with FigTree (v1.4.3) (http://tree.bio.ed.ac.uk/software/figtree/).

### 4.8. Prediction of MERS-CoV B Cell Epitopes

The IEDB [38] and BCPRED [39] servers were utilized to predict B cell epitopes of the MERS-CoV spike protein. IEDB software can predict both linear and discontinuous epitopes using a protein 3D structure (PDB format) as input. The software associates each epitope with its score, which is defined as a Protrusion Index (PI) method. For example, score value 0.8 means the predicted epitope would include within 80% of the protein amino acids and 20% of the protein amino acids will be outside of the epitope. Only epitopes that were located on the outer surface of the protein were chosen. Six residues in length was regarded as adequate to prompt a defensive immune reaction. We focused on discontinuous antigenic epitopes as they are increasingly explicit and have higher dominant attributes over linear epitopes [40,41].

## Figures and Tables

**Table 1:** List of viruses from genus Betacoronavirus used in this study showing their S protein and S gene homologous identities.

Organism Name	Accession Number	S Protein Identity (%)	S Gene Identity (%)
MERS-COV	MT226600 *	99.85	82.06
MERS-COV	MT226601 *	99.85	81.97
MERS-COV	MT226602 *	99.85	82.9
MERS-COV	MT226603 *	99.70	82.31
MERS-COV	MT226604 *	100.00	100.00
MERS-COV	MT226605 *	99.93	82.53
MERS-COV	MT226606 *	99.85	83.37
MERS-COV	MT226607 *	99.85	82.51
MERS-COV	KJ713297	99.78	82.46
MERS-COV	KY673149	99.63	82.36
MERS-COV	KF600612	99.78	82.16
MERS-COV	KF186564	99.78	83.00
MERS-COV	KF600652	99.78	82.31
MERS-COV	KF961222	99.78	82.71
MERS-COV	KF192507	99.70	83.74
2019-nCOV	EPI_ISL_416432	27.44	39.16
2019-nCOV	EPI_ISL_416522	27.44	40.43
2019-nCOV	NC_045512	27.44	40.46
2019-nCOV	MN988713.1	27.44	39.98
Batcoronavirus	KF636752.1	27.88	39.27
Batcoronavirus	GU190215.1	26.90	39.95
Batcoronavirus	DQ648857.1	26.99	39.42
Batcoronavirus	MK211374.1	26.94	39.27
Bat SARS coronavirus HKU3-12	GQ153547.1	66.32	65.78
Bat SARS-like coronavirus	KY417145.1	27.32	39.51
SARS-like coronavirus	KT444582.1	27.01	39.98
SARS-like coronavirus	AY278489.2	26.87	39.83
SARS coronavirus	NC_004718.3	26.87	39.22
SARS coronavirus	DQ497008.1	26.94	39.88
SARS coronavirus	FJ882957.1	26.94	39.44
SARS coronavirus	FJ882962.1	26.94	39.88

* Asterisks indicate MERS-CoV strains that were isolated in this investigation.

**Table 2:** Summary of predicted B cell epitopes present on the outer surface of the MERS-CoV, 2019-nCov, and SARS-CoV spike proteins.

Strain	Start	End	Peptide	Length	Score	3D Structure
MERS-CoV	523	566	YSPCVSIVPSTVWEDGDYYRKQLSPLEGGGWLVASGSTVAMTEQ	44	0.898	A
	702	743	STYGPLQTPVGCVLGLVNSSLFVEDCKLPLGQSLCALPDTPS	42	0.88	B
	485	518	PHNLTTITKPLKYSYINKCSRLLSDDRTEVPQLV	34	0.848	C
	90	99	GHATGTTPQK	10	0.839	D
	618	628	QNCTAVGVRQQ	11	0.827	E
	18	32	YVDVGPDSVKSACIE	15	0.801	F
2019-nCov	1071	1147	QEKNFTTAPAICHDGKAHFPREGVFVSNGTHWFVTQRNFYEPQIITTDNTFVSGNCDVVIGIVNNTVYDPLQPELDS	77	0.88	-
	92	192	FASTEKSNIIRGWIFGTTLDSKTQSLLIVNNATNVVIKVCEFQFCNDPFLGVNCTFEYVSFKNLREF	67	0.788	-
	328	364	RFPNITNLCPFGEVFNATRFASVYAWNRKRISNCVAD	37	0.767	-
	553	564	TESNKKFLPFQQ	12	0.721	-
SARS-CoV	684	702	DSSIAYSNNTIAIPTNFSI	19	0.712	-
	315	355	RFPNITNLCPFGEVFNATKFPSVYAWERKKISNCVADYSVL	41	0.692	-
	765	797	AQVKQMYKTPTLKYFGGFNFSQILPDPLKPTKR	33	0.638	-

**Table 3:** List of viruses from genus Betacoronavirus with sequences used in this study.

Organism Name	Host	Country	Accession Number
MERS-COV	*Camelus dromedarius*	Saudi Arabia	MT226600 *
MERS-COV	*Camelus dromedarius*	Saudi Arabia	MT226601 *
MERS-COV	*Camelus dromedarius*	Saudi Arabia	MT226602 *
MERS-COV	*Camelus dromedarius*	Saudi Arabia	MT226603 *
MERS-COV	*Camelus dromedarius*	Saudi Arabia	MT226604 *
MERS-COV	*Camelus dromedarius*	Saudi Arabia	MT226605 *
MERS-COV	*Camelus dromedarius*	Saudi Arabia	MT226606 *
MERS-COV	*Camelus dromedarius*	Saudi Arabia	MT226607 *
MERS-COV	*Camelus dromedarius*	Saudi Arabia	KJ713297
MERS-COV	*Camelus dromedarius*	Oman	KY673149
MERS-COV	*Homo sapiens*	Saudi Arabia	KF600612
MERS-COV	*Homo sapiens*	Saudi Arabia	KF186564
MERS-COV	*Homo sapiens*	Saudi Arabia	KF600652
MERS-COV	*Homo sapiens*	Qatar	KF961222
MERS-COV	*Homo sapiens*	United Arab Emirates	KF192507
2019-nCOV	*Homo sapiens*	Saudi Arabia	EPI_ISL_416432
2019-nCOV	*Homo sapiens*	Saudi Arabia	EPI_ISL_416522
2019-nCOV	*Homo sapiens*	China	NC_045512
2019-nCOV	*Homo sapiens*	USA	MN988713.1
Batcoronavirus	*Hipposideros pratti*	China	KF636752.1
Batcoronavirus	*Rhinolophus blasii*	China	GU190215.1
Batcoronavirus	*Rhinolophus macrotis*	China	DQ648857.1
Batcoronavirus	*Rhinolophus* sp.	China	MK211374.1
Bat SARS coronavirus HKU3-12	*Homo sapiens*	China	GQ153547.1
Bat SARS-like coronavirus	*Rhinilophus ferrumequinum*	China	KY417145.1
SARS-like coronavirus	*Rhinolophus sinicus*	China	KT444582.1
SARS-like coronavirus	*Homo sapiens*	China	AY278489.2
SARS coronavirus	*Homo sapiens*	Canada	NC_004718.3
SARS coronavirus	*Mus musculus*	USA	DQ497008.1
SARS coronavirus	*Mus musculus*	USA	FJ882957.1
SARS coronavirus	*Mus musculus*	USA	FJ882962.1

* Asterisks indicate MERS-CoV strains that were isolated in this investigation.

**Table 4:** Primer sets used for filling sequence gaps.

Primer Name	Sequence
Gap1_F1	CCTCGTTCTCTTGCAGAACTT
Gap1_R1	CCGGACGAAACCGTGTATT
Gap1_F2	CACTTCCCCTCGTTCTCTTG
Gap1_R2	ACCGTGTATTGTGACCGAGA
Gap2_F1	ACCAATTGGCTTATAGCTCTAGT
Cap2_R1	CCTTTAGGATCCGCCTCAAA
Gap3_F1	GGGATTACCCTAAGTGTGATAGAG
Gap3_R1	GTGCACTGACATTAGCAGTTG
Gap4_F1	CTGGGTTGTACCCAACCATT
Gap4_R1	CGTGCTGTAGGGTAGTAAATCG
Gap5_F1	TTTGCCAGGTTGTGATGG
Gap5_R1	ATACTCTCTGTACTCTGTAGCAT
Gap6_F1	TTCTTTACTTACCTGTGTAACCTCA
Gap6_R1	TCATAGGAGTGGAATTTCTCCAAA
Gap7_F1	CAGCATCAGCTCGTGATCTT
Gap7_R1	CTCCCAGAGCCTGATTAAACTTAT
Gap8_F1	CTCCTTTGGCCGTAGATGTT
Gap8_R1	CCGCTAGCAGGAATGTATGT
Gap8_F2	CCTTTGGCCGTAGATGTTGT
Gap8_R2	GCCGCTAGCAGGAATGTATG
Gap9_F1	AGATCGCGGCAATCGTT
Gap9_R1	GGCACTGTTCACTTGCAATC
